# Isolation and Characterisation of *in Vitro* and Cellular Free Radical Scavenging Peptides from Corn Peptide Fractions

**DOI:** 10.3390/molecules20023221

**Published:** 2015-02-16

**Authors:** Liying Wang, Long Ding, Ying Wang, Yan Zhang, Jingbo Liu

**Affiliations:** 1 Laboratory of Nutrition and Functional Food, Jilin University, Changchun 130062, China; E-Mails: wly625467126@163.com (L.W.); dinglong178@126.com (L.D.); jinkuang8499@163.com (Y.W.); zy01@jlu.edu.cn (Y.Z.); 2 College of Biological and Agricultural Engineering, Jilin University, Changchun 130062, China

**Keywords:** corn, peptide, antioxidant, free radical, amino acid, MADLI TOF/TOF MS

## Abstract

Corn gluten meal, a corn processing industry by-product, is a good source for the preparation of bioactive peptides due to its special amino acid composition. In the present study, the *in vitro* and cellular free radical scavenging activities of corn peptide fractions (CPFs) were investigated. Results indicated that CPF1 (molecular weight less than 1 kDa) and CPF2 (molecular weight between 1 and 3 kDa) exhibited good hydroxyl radical, superoxide anion radical and 2,2'-azino-bis (3-ethylbenzothiazoline-6-sulphonicacid) diammonium salt (ABTS) radical scavenging activity and oxygen radical absorbance capacity (ORAC). Meanwhile, the *in vitro* radical scavenging activity of CPF1 was slightly higher than that of CPF2. Both CPF1 and CPF2 also exhibited significant cytoprotective effects and intracellular reactive oxygen species scavenging activity in Caco-2 cells exposed to hydrogen peroxide (H_2_O_2_). The amino acid composition analysis revealed that the CPF were rich in hydrophobic amino acids, which comprised of more than 45% of total amino acids. An antioxidant peptide sequence of Tyr-Phe-Cys-Leu-Thr (YFCLT) was identified from CPF1 using matrix-assisted laser desorption/ionization time-of-flight/time-of-flight mass spectrometry (MALDI TOF/TOF MS). The YFCLT exhibited excellent ABTS radical scavenging activity with a 50% effective concentration (EC_50_) value of 37.63 µM, which was much lower than that of Trolox. In conclusion, corn gluten meal might be a good source to prepare antioxidant peptides.

## 1. Introduction

Reactive oxygen species (ROS), including free radicals such as superoxide, hydroxyl and non-radical species such as hydrogen peroxide (H_2_O_2_), are mainly generated from the respiratory chain of mitochondria and other sources under normal physiological conditions [1] and are involved in many biological systems as cell signaling molecules [2]. Therefore, a certain level of ROS is an essential component of living organisms and cells. However, when the body’s free radicals production exceeds their clearance capability, then oxidative stress will occur [3]. The accumulated intracellular ROS will attack large biomolecules and cell organs, increase lipid, protein and DNA oxidation, disturb cell membrane functions, and induce proteolysis and DNA mutations. This can promote aging and initiate several diseases, including cardiovascular disease, diabetes, cancer, rheumatoid arthritis and neurological disorders [4]. The free radical theory of ageing developed by Harman also speculated that the endogenous ROS were associated with aging and the degenerative diseases [5]. On the other hand, there are several intracellular biological antioxidant defense systems including enzymatic antioxidant system such as superoxide dismutase (SOD), catalase (CAT), glutathione peroxidase (GP-x) and non-enzymatic antioxidant system such as glutathione (GSH). In addition, some antioxidants such as ascorbic acid, tocopherol, flavonoids, also help to clear free radicals. However, these molecules are often not enough to counteract the excessive ROS in living organism especially when oxidative stress occurs. Therefore, some exogenous antioxidants provided by food are also critical to keep the oxidation-reduction balance [6].

Compared to synthetic antioxidants, natural compounds derived from foods not only show high antioxidant properties but also little side effects. Particularly, bioactive peptides derived from food protein have been found to possess considerable antioxidant capacities [7]. Therefore, food-derived antioxidant peptides have been widely studied and a great number of antioxidant peptides were identified since Marcuse first reported the antioxidant properties of some amino acids in 1960 [8]. Different from animal proteins, plant proteins are becoming an interesting source for bioactive peptides due to their low price and high abundance [9]. For instance, soybean [10], corn [11], barley [12], wheat [13], rice bran [14], peanut [15], chickpea [16], potato [17], flaxseed [18] have all been demonstrated to be good protein sources for preparation of antioxidant peptides.

Corn gluten meal mainly comprises 65% (w/w) zein and 30% (w/w) glutelin [9]. It is a by-product of the corn processing industry usually used as forage due to its poor nutritional quality, *i.e.*, less digestibility and lack of essential amino acids such as lysine and tryptophan [19]. Nevertheless, corn gluten meal has a high level of hydrophobic amino acids (HAA) and branched chain amino acids (BCAA) [20]. This makes corn gluten meal a good source for preparation of bioactive peptides, especially for hepatoprotective peptides [21,22], Angiotensin-converting enzyme (ACE) inhibitory peptides [23,24] and antioxidant peptides [9,11,25,26,27]. Orally administrated proteins or peptides are digested and degraded by extensive enzymatic degradation systems in gastrointestinal tract before they are absorbed in the intestinal epithelium [28]. Studies in Caco-2 cell monolayer systems, a good intestinal model for the absorption of drugs firstly described systematically by Hidalgo [29], have shown that the size and the lipophilicity of peptides are two critical parameters in determining their permeability through the intestinal epithelium [30,31]. Low molecular weight and hydrophobic peptides are absorbed more easily in an intact form. However, there are few studies on the low molecular weight antioxidant peptides derived from corn gluten meal.

The objective of the present study was to investigate the *in vitro* and cellular free radical scavenging activities of low molecular weight CPF derived from corn gluten meal hydrolysate. Furthermore, the amino acid compositions of CPF were determined. An antioxidant peptide sequence of YFCLT was identified and further synthesized to validate its antioxidant activities.

## 2. Results and Discussion

### 2.1. In Vitro Free Radical Scavenging Activities of CPF

In the present study, the antioxidant activities of CPF were evaluated by the methods based on the *in vitro* free radical scavenging capacity, for instance, hydroxyl radical scavenging activity, superoxide anion radical scavenging activity, ABTS radical scavenging activity and oxygen radical absorbance capacity (ORAC) assay.

**Figure 1:**
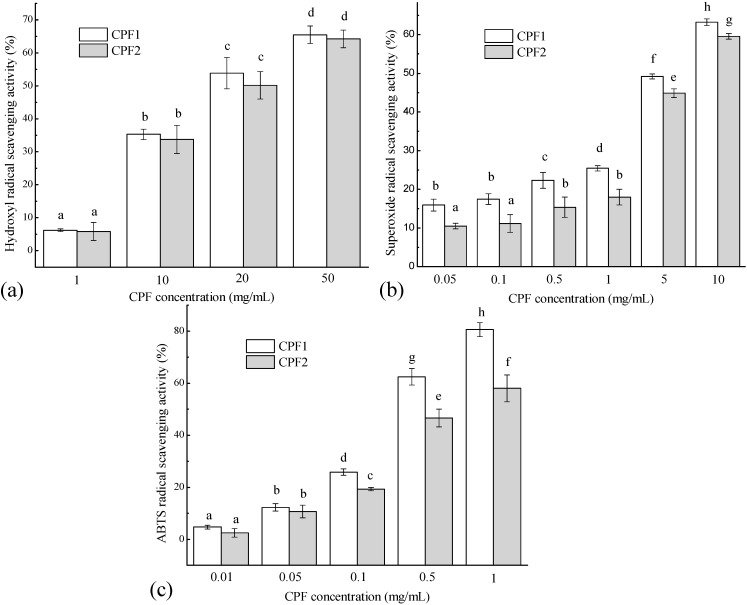
*In vitro* free radical scavenging activities of CPF fractions. (**a**) Hydroxyl radical scavenging activity; (**b**) Superoxide anion radical scavenging activity; (**c**) ABTS radical scavenging activity. For each measurement, the data marked by different letters are significantly different (*p* < 0.05).

Hydroxyl radicals are the most reactive species, which can react with biomolecules, such as amino acids, proteins and DNA, and then induce severe damages, such as protein or lipid peroxidation, DNA mutation in cells [32]. The hydroxyl radicals were generated by inducement of the ferrous ion in a Fenton reaction in the present study. Results revealed that CPF showed significant hydroxyl radical scavenging activity in a concentration-dependent way. At the final concentration of 50 mg/mL, CPF1 and CPF2 exhibited 65.47% ± 2.66% and 64.26% ± 2.69% scavenging effect on hydroxyl radicals with EC_50_ values of 17.14 and 19.86 mg/mL, respectively (Figure 1a). Different from hydroxyl radical, superoxide anion radicals are not highly reactive, but are precursors of other highly reactive species, such as hydroxyl radicals and hydrogen peroxide. Both superoxide anion radicals and their derivatives are cell-damaging, which can cause damage to the DNA and membrane of cells [33]. In the present study, the CPF also exhibited good superoxide anion radicals scavenging activities in a broad range when CPF concentration increased from 0.05 to 10 mg/mL (Figure 1b). The highest superoxide anion radicals scavenging activities of CPF1 and CPF2 were 59.58% ± 0.72% and 63.22% ± 0.88% (EC_50_ 6.12 and 5.10 mg/mL, respectively) when the concentration of 10 mg/mL was used. In addition, CPF1 showed slightly higher hydroxyl radicals and superoxide anion radicals scavenging activities than CPF2.

The ABTS radical scavenging assay can be applied to both lipophilic and hydrophilic compounds. It is also excellent in evaluating antioxidant capacity of protein hydrolysates and peptides, in which peptide fractions can act as electron or hydrogen donors in free radicals reactions [34,35]. As shown in Figure 1c, the effects of CPF on ABTS radical scavenging activities were analysed and the EC_50_ values of 0.34 and 0.54 mg/mL were measured for CPF1 and CPF2, respectively. Overall, CPF1 exhibited higher ABTS radical scavenging activity than CPF2. Especially, at the highest concentration of 1 mg/mL, CPF1 scavenged 80.63% ± 2.69% radicals (Trolox equivalent antioxidant capacity (TEAC)): 174.72 ± 5.98 µM TE/g peptide), which was higher than 57.99% ± 5.17% (TEAC: 124.41 ± 11.48 µM TE/g peptide) of CPF2 (*p* < 0.01). These results demonstrated the positive effects of CPF1 and CPF2 fractions on the ABTS radical scavenging activities and a higher scavenging activity of CPF1.

**Figure 2:**
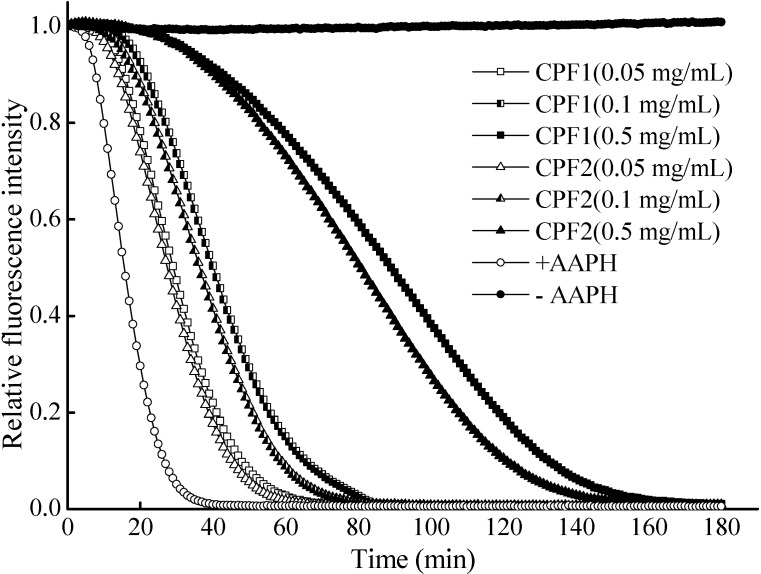
Radical scavenging activity of CPF toward peroxyl radicals (ORAC assay). +AAPH represented no CPF was added, but the AAPH was normally used. –AAPH respresented not only no CPF was added, but also no AAPH was used.

The ORAC assay was further used to evaluate the free radical scavenging activities of CPF to quench peroxyl radicals generated by 2,2-azo-bis (2-methylpropionamidine) dihydrochloride (AAPH) and protect the fluorescein. The protective effect was measured by assessing the fluorescence decay curve [7]. Figure 2 depicted the effects of CPF on the time-dependent decay of fluorescein induced by AAPH. The result showed that both CPF exhibited a concentration-dependent increase in the inhibition of fluorescein decay. When the concentration of CPF was 0.5 mg/mL, the ORAC values of CPF1 and CPF2 fractions were 935.43 ± 28.10 and 833.34 ± 29.29 µM TE/g peptide, respectively.

The *in vitro* free radical scavenging assay is directly, easily and widely used to evaluate the antioxidant properties of compounds. In the present study, it was found that the *in vitro* free radical scavenging activities of CPF (molecular weight less than 1 kDa and 1–3 kDa), particularly the ABTS radical scavenging activity, were normally higher than that reported by Zhuang *et al.* [36]. The ORAC value of CPF in this study was also much higher than that reported by Zhou *et al.*, in which corn protein was hydrolysed by three different proteases [27]. In addition, Li *et al*., obtained the CPF with different molecular weight, which showed excellent hydroxyl radical and superoxide anion radical scavenging activities [25]. Furthermore, it was found that the order of *in vitro* free radical scavenging activity of CPF was ORAC > ABTS radical scavenging activity > superoxide anion radical scavenging activity > hydroxyl radicals scavenging activity in the present study. This may due to the differences among different antioxidant access systems. However, the relatively low hydroxyl radicals scavenging activity of CPF was still comparable to the antioxidant peptides derived from loach protein hydrolysates reported by You *et al.* [37]. Overall, the CPF prepared in the present study exhibited good *in vitro* free radical scavenging properties.

### 2.2. Cytoprotective Effects of CPF on Caco-2 Cells Exposed to H_2_O_2_

**Figure 3:**
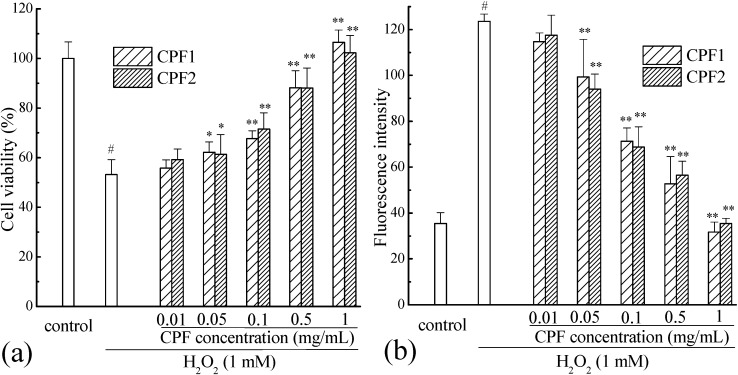
Cytoprotective effect (**a**) and intracellular ROS scavenging capacity (**b**) of CPF on Caco-2 cells from oxidative stress induced by H_2_O_2_. # *p* < 0.001 compared to control group, * *p* < 0.01 compared to damage group (only H_2_O_2_ but no CPF treated group), ** *p* < 0.001 compared to damage group (only H_2_O_2_ but no CPF treated group).

As shown in Figure 3a, the viability of Caco-2 cells decreased significantly to 53.15% ± 6.09% (*p* < 0.001). However, pretreatment with CPF improved the Caco-2 cells viabilities with a significant dose-response effect (*p* < 0.001). The CPF at low concentration of 0.01 mg/mL showed no cytoprotective effect on H_2_O_2_ induced Caco-2 cells (*p* > 0.05), but at the concentration of 0.05 mg/mL, both CPF1 and CPF2 significantly improved the cell viabilities up to 62.20% ± 4.13% and 61.32% ± 7.99%, respectively (*p* < 0.01). When the concentrations increased to 1 mg/mL, CPF1 and CPF2 showed the strongest protection by enhancing cell viabilities up to 106.48% ± 5.02% and 102.33% ± 6.98%, respectively (*p* < 0.001). All these results suggested that the CPF exhibited significant cytoprotective effect on Caco-2 cells from oxidative damage induced by H_2_O_2_. This may be due to that CPF can not only scavenge the intracellular ROS directly, but also activate some signaling pathways related to the cellular defense system against oxidative stress [2].

### 2.3. Intracellular ROS Clearance Capacity of CPF

Once 2',7'-dichlorofluorescin di-acetate (DCFH-DA) diffuses into the cell, it undergoes an enzymatic degradation by cellular esterases to form DCFH. Then, in the presence of the accumulated intracellular ROS induced by H_2_O_2_, DCFH is usually oxidized to highly fluorescent DCF, which can be detected by a fluorescent reader. The antioxidant property of CPF was evaluated as the reduce level of ROS induced by H_2_O_2_. As shown in Figure 3b, the ROS level of H_2_O_2_ group was more than 3 times that of control group, indicating that the accumulated intracellular ROS increased significantly after H_2_O_2_ induction (*p* < 0.001). Interestingly, besides the cytoprotective effect on Caco-2 cells exposed to H_2_O_2_, pretreatment with CPF could also exhibit good intracellular ROS clearance capacity with significant dose-response effect (*p* < 0.001). This result was consistent with the cytoprotective effect. A low concentration of 0.01 mg/mL of CPF decreased the intracellular ROS level slightly, but without significance (*p* > 0.05). However, the intracellular ROS levels of the high concentration CPF-pretreated groups were significantly lower than that of H_2_O_2_-induced damage group (*p* < 0.001). When the concentration increased up to 1 mg/mL, the intracellular ROS level induced by H_2_O_2_ was sharply reduced (*p* < 0.001) and it was similar to that of control group (*p* > 0.05).

Under normal conditions, cellular antioxidant defense systems including enzymatic and non-enzymatic antioxidants play a major role in removing the cellular accumulated ROS, but fail to protect cells from oxidative damage induced by excess ROS. The cellular response to oxidative stress has not yet been completely understood. This may involve, on one hand, increasing the levels of cellular antioxidant defenses, and on the other hand, adapting to the stress based on ROS-dependent signaling pathways [38]. It has been widely accepted that nuclear factor-kappa B (NF-κB) and activator protein-1 (AP-1) can be activated by oxidative stress and ROS to regulate the gene expression and protein synthesis associated with cellular defense [39,40]. Recent researches have indicated that a new and a critical transcription factor nuclear factor erythroid 2-related factor 2 (Nrf2) can penetrate nucleus and bind to the antioxidant response elements (ARE), then initiate the transcription of the down downstream target genes for antioxidant enzymes such as SOD, CAT, GP-x, NADP(H):quinone oxidoreductase 1 (NQO1), and cytoprotective proteins such as heme oxygenase 1 (HO1) [41]. However, it should be realized that some pitfalls of the oxidative stress model exist and further *in vivo* animal experiments are needed [38]. Therefore, to identify the molecular mechanism of intracellular ROS scavenging capacity of CPF, more experiments should be conducted in the future.

### 2.4. Amino Acid Composition of CPF

The bioactive activities of CPF might be highly related to their amino acid composition. Thus, the amino acid composition of CPF1 and CPF2 fractions was determined and analysed. As shown in Table 1, the total amino acids contents (eighteen normal amino acids) in CPF1 and CPF2 were 63.07 and 68.78 g amino acid residues/100 g CPF powder, respectively.

**Table 1:** Amino acid composition of CPF.

Amino Acid	CPF1		CPF2	
	Concentration	Composition	Concentration	Composition
	(g Amino Acid/kg CPF)	(%)	(g Amino Acid/kg CPF)	(%)
Asp	38.0	6.03	42.3	6.15
Thr	19.9	3.16	23.0	3.34
Ser	30.0	4.76	32.7	4.75
Glu	142.1	22.53	149.4	21.72
Gly	19.3	3.06	22.1	3.21
Ala	49.0	7.77	46.0	6.69
Val	28.4	4.50	36.1	5.25
Met	16.2	2.57	17.0	2.47
Ile	22.9	3.63	26.1	3.79
Leu	96.8	15.35	92.7	13.48
Tyr	33.7	5.34	35.0	5.09
Phe	37.9	6.01	36.6	5.32
His	13.4	2.12	14.8	2.15
Lys	10.4	1.65	17.2	2.50
Arg	15.2	2.41	15.8	2.30
Pro	46.4	7.36	63.5	9.23
Trp	1.3	0.21	1.5	0.22
Cys	9.8	1.55	16.0	2.33
THAA ^a^	298.9	47.40	319.5	46.45
BCAA ^b^	148.1	23.48	154.9	22.52
Total	630.7	100	687.8	100

^a^ THAA, the total hydrophobic amino acids, including Ala, Val, Met, Ile, Leu, Phe, Pro and Trp; ^b^ BCAA, the branched chain amino acids, including Leu, Ile and Val.

It has been found that several amino acids such as His, Tyr, Met, Leu, Trp and Lys are generally accepted as antioxidants which contribute to the scavenging of free radicals [42,43]. In the present study, the amount of these six antioxidant amino acids were 20.46 g/100 g CPF (32.44%) and 20.72 g/100 g CPF (30.13%) in CPF1 and CPF2, respectively. In addition, the CPF both contained high level of total hydrophobic amino acid (THAA) such as Ala, Val, Met, Ile, Lue, Phe, Pro and Typ (29.89 g/100 g CPF (47.4%) and 31.95 g/100 g CPF (46.45%) for CPF1 and CPF2, respectively), which has been demonstrated to play a critical role in the antioxidant effect [44,45,46]. Therefore, the abundance of antioxidant amino acids might explain why CPF1 and CPF2 fractions possessed high free radicals scavenging abilities. Interestingly, CPF1 had slightly higher proportion of the above two kinds of potential antioxidant amino acids than CPF2, which might be the cause of why CPF1 exhibited slightly higher *in vitro* free radicals scavenging activity than CPF2. On the other hand, CPF1 with low molecular weight contained more small peptides and *N*-terminal and *C*-terminal amino acids residues, which were also thought to be very important for antioxidant properties. However, these highlights had not been validated by the result of the intracellular ROS scavenging activity.

### 2.5. Characterization of Antioxidant Peptide from CPF

**Figure 4:**
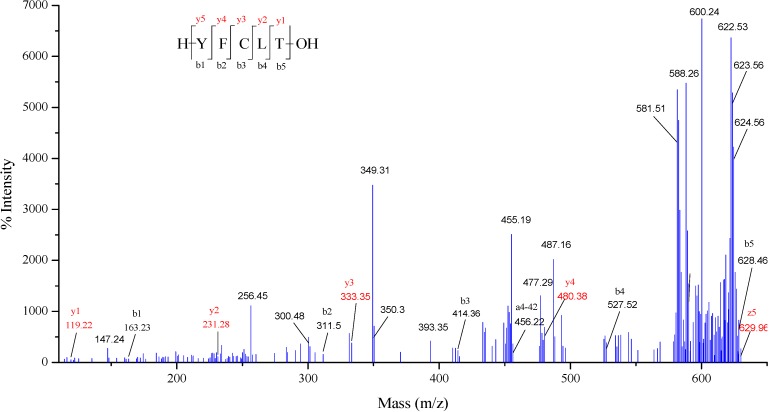
Identification of antioxidant peptide: MS/MS spectra of singly charged ion with *m/z* 644.07 was determined to be the peptide sequence of YFCLT.

**Figure 5:**
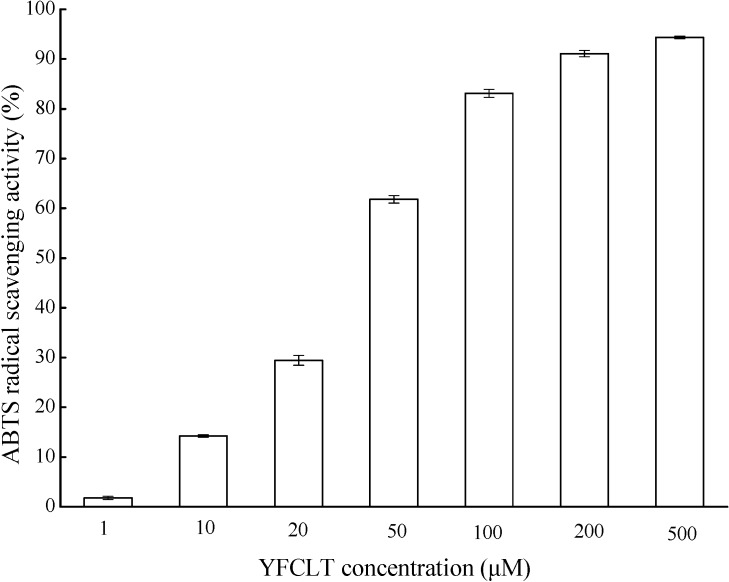
ABTS radical scavenging activity of peptide YFCLT.

The molecular weight and amino acid sequence of CPF1 was identified by MALDI-TOF/TOF mass spectroscopy. MS/MS spectra of a single-charged ion with *m/z* at 644.07 Da is shown in Figure 4. This peptide comprised five amino acid residues and its amino acid sequence was identified to be YFCLT (Tyr-Phe-Cys-Leu-Thr), which was a new peptide that had not been previously reported. The spectra of YFCLT contained the complete series of fragment b ions and mostly series of fragment y ions, which were in agreement with the collision-induced dissociation (CID) that mainly produces the b and y ions. The losses of precursor ion (*m/z* at 644.07 Da) and the presence of major z ion on *C*-terminal (*m/z* at 629.96 Da) were usually caused by the high collision energy. In addition, it was commonly difficult to distinguish the isobaric amino acids Leu and Ile, which had same b-type fragments. Johnson *et al.* had reported that Leu could be distinguished from Ile using a_n_ ion types on *N*-terminal fragments, namely, a_n_-42 for Leu and a_n_-28 for Ile [47]. Thus, a Leu residue was confirmed in the antioxidant peptide sequence. In addition, a peptide sequence of GDCPCR (Gly-Asp-Cys-Pro-Cys-Arg) was also identified (data not show). Subsequently, the identified peptide of YFCLT and GDCPCR were synthesized by the 9-fluorenylmethoxycarbonyloxy (Fmoc) protected amino acids synthetic methods, and its antioxidant property was measured using the ABTS radical scavenging assay. However, only YFCLT exhibited significant free radical scavenging activity. As shown in Figure 5, YFCLT exhibited excellent ABTS radical scavenging activity with an EC_50_ value of 37.63 µM, which was much lower than that of Trolox. Nevertheless, what should be noted is that CPF1 may contain many peptides with antioxidant activity and YFCLT is just one of them. Further works are still needed to identify antioxidant peptide from CPF.

The antioxidant properties of peptides may dependent on their amino acid composition, structure, hydrophobicity, and other factors. The antioxidant peptide YFCLT contains two hydrophobicity amino acid residues such as Phe and Leu, which usually exist in antioxidant peptides. This peptide sequence also contains two aromatic residues Tyr and Phe, which may play critical roles as effective free radical scavengers. That is because aromatic amino acids can donate protons to electron deficient radicals constantly [48]. Furthermore, the presence of Cys residue in the center of the antioxidant peptide can also facilitate its free radicals scavenging properties since the thiol group in Cys residue can interact with radicals directly [49]. Thus, all these special amino acid residues might contribute to the high free radical scavenging activity of peptide YFCLT.

## 3. Experimental Section

### 3.1. Materials

Corn gluten meal was obtained from Dacheng Ltd. (Changchun, China). Caco-2 cell lines were purchased from the Chinese Academy of Sciences Type Culture Collection (Shanghai, China). The peptide YFCLT with a purity of 95% was synthesized by ChinaPeptides Co., Ltd. (Shanghai, China). Alcalase was purchased from Novo Co. (Novo Nordisk, Copenhagen, Denmark). 1,1-Diphenyl-2-picrylhydrazyl (DPPH), 1,10-phenanthroline, ethylenediamine tetraacetate (EDTA), 2,2'-azino-bis(3-ethylbenzothiazoline-6-sulphonicacid) diammonium salt (ABTS), 2,2-azo-bis(2-methylpropionamidine) dihydrochloride (AAPH), 2',7'-dichlorofluorescin diacetate (DCFH-DA), 6-hydroxy-2,5,7,8-tetramethylchroman-2-carboxylicacid (Trolox), ferrous sulfate, Tris-HCl, potassium persulfate, fluorescein, vitamin C, pyrogallol were purchased from Sigma-Adrich (St. Louis, MO, USA). Dulbeco’s Modified Eagle’s Medium (DMEM), fetal bovine serum, penicillin-streptomycin, phosphate buffered solution (PBS) were purchased from Gibco BRL Life Technology (Carlsbad, CA, USA). 3-(4,5-Dimethylthiazol-2-yl)-5-(3-carboxymethoxyphenyl)-2-(4-sulfophenyl)-2H-tetrazolium (MTS) was purchased from Promega Biotechnology Co. Ltd. (Madison, WI, USA). Hydrogen peroxide (H_2_O_2_) was purchased from Alfa Aesar (Ward Hill, MA, USA). All the other reagents were of analytical grade.

### 3.2. Corn Gluten Peptide Preparation

Corn gluten meal was crushed and sieved using a 120 mesh sieve. The permeate was collected and dispersed in distilled water to obtain 3.3% protein slurry (w/v) in a 50 L reactor, followed by heating to 90 °C for 10 min in order to denature the protein. When the mixture was cooled down to 60 °C, 1 M sodium hydroxide was used to adjust the pH to 10. Then, the mixture was hydrolysed by alkaline proteinase Alcalase using an enzyme/substrate ratio of 9% for 3 h under continuous mixing. At the end of the hydrolysis period the mixture was heated to 90 °C for 10 min to inactivate the protease. The hydrolysate was centrifuged at 4000 r/min for 10 min and the supernatants were fractionated using ultrafiltration with molecular weight cut-off (MWCO) membranes of 3 and 1 kDa (Millipore, Billerica, MA, USA). Two fractions were obtained and designed as follows: CPF1 (molecular weight less than 1 kDa), CPF2 (molecular weight between 1 and 3 kDa), then they were lyophilised and stored at −20 °C until use.

### 3.3. Determination of in Vitro Free Radical Scavenging Activities of CPF

#### 3.3.1. Hydroxyl Radical Scavenging Activity Assay

The hydroxyl radical scavenging activity of CPF was performed according to the method described by Zhu *et al.* [50]. Thirty microliters of sample solution at different concentrations (1–50 mg/mL) was mixed with 50 µL of 1,10-phenanthroline (5.0 mM), 50 µL of FeSO_4_ (5.0 mM), 50 µL of EDTA (15 mM) and 30 µL of sodium phosphate buffer (0.2 M, pH 7.4) in a 96-well microplate. After mixing, 60 µL of H_2_O_2_ (0.1%) was added to initiate the Fenton reaction. The reaction mixture was incubated for 1 h at 37 °C, and the absorbance was measured at 536 nm using a microplate reader (BioTek Instruments, Winooski, VT, USA). Damaged group contained the same solutions as sample group except using distilled water instead of sample. Non-damaged group contained the same solutions as damaged group except using distilled water instead of H_2_O_2_. The hydroxyl radical scavenging activity was calculated as follows:

The hydroxyl radical scavenging activity (%) = (A_sample_ − A_damage_)/(A_non-damage_ − A_damage_) × 100

#### 3.3.2. Superoxide Anion Radical Scavenging Activity Assay

The superoxide anion radical scavenging activity of CPF was determined by the method of Bamdad *et al.* [51]. This assay is dependent on the reducing activity of antioxidant by an superoxide anion radical dependent reaction, which releases chromophoric products. Briefly, 80 µL of sample solution at different concentrations (0.05–10 mg/mL) was mixed with 80 µL of 50 mM Tris-HCl buffer (pH 8.3) containing 1 mM EDTA in a 96-well microplate and 40 µL of 1.5 mM pyrogallol in 10 mM HCl. The rate of superoxide anion radical-induced polymerization of pyrogallol (ΔA/min) was measured as an increase in absorbance at 320 nm for 5 min at 23 °C using a microplate reader. Tris-HCl buffer was used instead of sample as control. The superoxide anion radical scavenging activity was calculated as follows:

The superoxide anion radical scavenging activity (%) = (ΔA_control_/min − ΔA_sample_/min)/ΔA_control_ × 100

#### 3.3.3. ABTS Radical Scavenging Activity Assay

The ABTS radical scavenging assay was performed by the method of Hernandez-Ledesma *et al.* [52]. ABTS^+^ was produced by reaction of 7 mM ABTS solution and 2.45 mM potassium persulfate (final concentration) in the dark at room temperature for 12–16 h before use. The ABTS^+^ solution was adjusted to an absorbance of 0.70 ± 0.02 at 734 nm on a spectrophotometer (UV-2550, Shimadzu, Kyoto, Japan) by dilution with 5 mM phosphate buffered solution. An aliquot of 50 µL of sample solution at different concentrations (0.01–1 mg/mL) was added to 150 µL of the diluted ABTS^+^ solution, followed by incubation for 10 min at room temperature. The absorbance of the mixture at 734 nm was measured. The ABTS scavenging activity was calculated as follows:

ABTS radical scavenging activity (%) = (A_control_ − A_sample_)/A_control_ × 100

The ABTS scavenging activity of the sample was expressed as Trolox Equivalent Antioxidant Capacity (TEAC, µM TE/g sample).

#### 3.3.4. Oxygen Radical Absorbance Capacity (ORAC) Assay

ORAC assay was investigated as previously described by Ou *et al.* [53] and Davalos *et al.* [54]. The reaction was conducted in 75 mM sodium phosphate buffer (pH 7.4) in black 96-well microplate. Briefly, an aliquot of 20 µL of sample was added to 120 µL of fluorescein solution (200 nM), followed by incubation for 3 min at room temperature. Then, 60 µL of AAPH (130 mM) was added to the mixture. The fluorescence was recorded every 1 min using a microplate reader with 485-P excitation and 520-P emission filters. Sodium phosphate buffer and Trolox were used as control and standard sample, respectively. Fluorescence measurements were normalized to the curve of the blank. From the normalized curves, the area under the fluorescence decay curve (AUC) was calculated as follows:

$$AUC=1+\sum_{i=0}^{180}f_{i}/f_{0}$$

where f_o_ and f_i_ were the fluorescence reading at time 0 min and i min, respectively. The net AUC corresponding to a sample was calculated as follows:

net AUC = AUC_antioxidant_ − AUC_blank_

The antioxidant ORAC value was calculated by a linear regression analysis between net AUC and antioxidant concentration and expressed as µM TE/g sample.

### 3.4. Cell Culture

The Caco-2 cells were grown in DMEM containing 10% fetal bovine serum, 1% nonessential amino acid solution, 100 units/mL penicillin, 100 µg/mL streptomycin and 4 mM L-glutamine at 37 °C in an atmosphere of 5% CO_2_ and 90% relative humidity. Stock cultures were grown in 75 cm^2^ tissue culture flasks and were split at 80% to 90% confluency using 0.25% trypsin and 0.02% EDTA solution. The cells from passage numbers of 30 to 40 were used and oxidative stress was induced as described by Katayama and Young [55,56]. Namely, Caco-2 cells were seeded in 96-well culture plates (at a density of 5 × 10^3^ cells/well) for 24 h. CPF with different concentrations dissolved in PBS were added to cells and incubated for 2 h followed by addition of 1 mM H_2_O_2_ (final concentration) solution for another 6 h of incubation time. The cell viability of Caco-2 cell was determined using the MTS assay [57]. Briefly, MTS was added in the plates (20 µL MTS per 100 µL culture medium was used) and incubated at 37 °C in 5% CO_2_ for 2 h followed by detection the absorbance at 490 nm using a microplate reader.

### 3.5. Determination of Intracellular ROS Scavenging Activities of CPF

Intracellular ROS level was determined using DCFH-DA fluorescent probe according to the method described by LeBel *et al*. [58] with some modifications. The pretreatment was conducted as described above, namely, Caco-2 cells was incubated with different concentrations of CPF for 2 h and then incubated with 1 mM H_2_O_2_ solution for 6 h. Then, cell culture medium was moved out and the Caco-2 cells were washed with PBS. An aliquot of 20 µL of DCFH-DA fluorescent probe solution was added to cells and incubated for 20 min at 37 °C in 5% CO_2_ and then washed twice with PBS. Finally the fluorescence intensity of DCF was measured with an excitation wavelength of 485 nm and an emission wavelength of 530 nm using a microplate reader.

### 3.6. Amino Acid Analysis

The amino acid analysis was performed according to the Chinese National Standard Method (Chinese Standard GB/T 18246-2000 and GB/T 15400-1994) [59,60] with some modifications. Briefly, the sample of CPF was hydrolysed with 6 M HCl at 110 °C for 24 h prior to cation exchange separation and derivatization with ninhydrin, except for that alkaline hydrolysis was used to determine the tryptophan amount. The amino acid composition of CPF was determined using an automatic amino acid analyzer (Sykam Amino Acid Analyzer S 433D, Munich, Germany).

### 3.7. Identification of Antioxidant Peptide by MALDI-TOF/TOF MS/MS

The obtained CPF were desalted using ZipTips (Millipore, Billerica, MA, USA) and analysed using a 5800 MALDI-TOF/TOF mass spectrometer (AB Sciex, Framingham, MA, USA) with a 1000 Hz high-repetition OptiBeam^TM^ on-axis laser and a QuanTis^TM^ precursor ion selector according to the method of Zhang *et al*., with some modifications [61]. The peptide was mixed with the matrix solution (50% acetonitrile and 0.1% TFA), and 0.5 µL of the mixture was spotted on the MALDI target plate. The MALDI-TOF/TOF MS/MS analysis was run in the positive reflector mode at 20 kV collision induced dissociation (CID) using air as the collision gas.

### 3.8. Data Analysis

All data were expressed as the mean ± SD (*n* = 3). The difference was carried out by one-way analysis of variance (ANOVA) with the significance level at *p* < 0.05 by SPSS 19.0 software.

## 4. Conclusions

In the present study, the obtained CPF (MW less than 1 kDa and 1–3 kDa) exhibited hydroxyl radical scavenging activity, superoxide anion radical scavenging activity, ABTS radical scavenging activity and oxygen radical absorbance capacity. In addition, the CPF also exhibited significant cytoprotective effect and intracellular ROS scavenging activity in Caco-2 cells exposed to H_2_O_2_. The CPF contained large number of hydrophobic amino acids (more than 45%) which may contribute to their antioxidant properties. Peptide sequence of YFCLT was indentified using MADLI TOF/TOF MS from CPF1. ABTS radical scavenging activity assay showed that YFCLT exhibited excellent ABTS radical scavenging activity with an EC_50_ value of 37.63 µM, which was much lower than that of Trolox.
